# Behavioural Risk for HIV, Hepatitis B, and Hepatitis C Infections among a Population of Drug Users and Injectors across Four Regions in Ghana

**DOI:** 10.1155/2022/2544481

**Published:** 2022-09-01

**Authors:** Chris Guure, Sharren Margaret Obiri-Yeboah Laryea, Samuel Dery, Carlota Baptista da Silva, Comfort Asamoah-Adu, Stephen Ayisi-Addo, Maria-Goretti Loglo, Adamu Mohammed, Kwasi Torpey

**Affiliations:** ^1^Department of Biostatistics, School of Public Health, University of Ghana, Legon, Accra, Ghana; ^2^International Consultant, Harm Reduction and Key Population Expert, Lisbon, Portugal; ^3^West Africa Program to Combat AIDS, Accra, Ghana; ^4^National AIDS/STI Control Programme, Ghana Health Service, Accra, Ghana; ^5^International Drug Policy Consortium, East Legon, Accra, Ghana; ^6^West Africa Behavioural Health Addictions and Recovery Management, Accra, Ghana; ^7^Department of Population Family and Reproductive Health School of Public Health, University of Ghana, Legon, Accra, Ghana

## Abstract

**Background:**

Blood borne infections such as HIV, Hepatitis B (HBV), and Hepatitis C (HCV) are of great importance to governments and their implementing partners, especially among people who use drugs (PWUD) and people who inject drugs (PWID). Prevalence and determinants of HIV, HBV, and HCV among PWUD and PWID in Ghana are not well established, the significance of this study.

**Method:**

This assessment was a cross-sectional study implemented via the respondent driven sampling approach. A team of community advisory boards that comprised former users, current users, and civil society organizations were constituted to help in the implementation of the study. The study was conducted in four regions in Ghana. The assessment was based on a representation of populations of PWID and PWUD from the four regions. Efforts were made by the team to ensure adequate representation of women where feasible. A quantitative questionnaire was developed and used to obtain information on the respondents' sociodemographics, sexual behavior, substance use, and biological characteristics. The prevalence of HIV, HBV, and HCV among PWID and PWUD was determined using blood samples. First response and oral quick test for confirmation of HIV positivity were carried out, while SD bioline was used to test for the presence of HBV and HBC. Data were analyzed using the Bayesian generalized linear model via the binomial family of distributions under the logit link function with weak Cauchy and Normal distribution as prior.

**Results:**

A total of 323 PWUD and PWID participants were interviewed across four regions of Ghana. The overall median age of the respondents was 36 (28, 43) years. The prevalence of HIV, HBV, and HCV infection in the study was 2.5%, 4.6%, and 5.9%, respectively. The prevalence of HIV, HBV, and HCV among drug users was 2.5% (95% CI: 0.7%–4.2%), 4.1% (95% CI: 1.8%–6.2%), and 6.7% (95% CI: 3.9%–9.4%), respectively. Most drug injectors and users started using and injecting drugs at ages less than 20 years and between 20 and 29 years, respectively. Drug users who identified themselves as part of the general population were 66% less likely to be tested HIV positive (POR = 0.34, CrI: 0.12–0.81) compared to sex workers. Part time employment respondents had fivefold odds (POR = 5.50, CrI: 1.20–16.16) of being HBV positive as against full-time employment.

**Conclusion:**

Most of the injectors and users started drugs at an early age. Drug users and injectors are at higher risk of these infections because of associated risky sexual behaviors and risky injection practices. Harm reduction programs to help addicts who are willing to quit the practice are recommended.

## 1. Background

Blood-borne infections such as HIV, Hepatitis B, and C are of great significance to public health experts [1]. These diseases have rapidly gained grounds among drug users and drug injectors, increasing their contribution to the global burden of these diseases [2–6]). Globally, 11,180,000 people inject drugs, and in the year 2018, there was a 30% increase in drug users from the previous decade to an estimated 270 million. As of 2020, more than 35 million drug users experienced substance use disorders. With the advent of the COVID-19 pandemic, fallouts in the global economy have led to joblessness, contributing to the increased numbers of drug users [6, 7]. Moreover, the COVID-19 impact on international commerce and drug scarcity has led to the increased cost of drugs and loss of drug purity, hence, the increase in more detrimental drug use patterns such as the use of synthetic drugs and drug injection [7]. Among drug injectors worldwide, the prevalence of HIV, hepatitis B, and C in the year 2021 were 12%, 8.7%, and 50%, respectively, with the ages of drug injectors spanning from 12 to 65 years. Other studies also suggest a significantly higher prevalence of HIV and hepatitis C among drug users compared to the general population, although injection drug users are at more risk of contracting these blood-borne infections, including hepatitis B, as opposed to drug users [8, 9]. This is usually because of the high communal use of injection accessories such as cotton, cookers, water, and needles [10, 11]. Among noninjecting drug users, the risk of HIV, hepatitis B, and C is mainly due to the link between drug use and risky sexual behaviour [12].

Africa is home to about 950,000 drug injectors. Out of this number, HIV, hepatitis B, and C infected drug injectors were 10.9%, 6.8%, and 19.9% of the population of drug injectors [7]. In Ghana, most drug users are of a low socioeconomic class and usually combine drugs such as marijuana, cocaine, and heroin (which are normally smoked than injected) [13]. In 2013, it was found that the prevalence of HIV among drug injectors (4%) in Ghana was found to be lower than the global figure (10%) in 2013. The few who injected drugs were shunned by the local community of drug users as it was seen as a foreign practice. It is speculated that drug injectors acquired the habit when they traveled outside the country [13]. A 2021 study conducted in Ghana, however, indicates a recent surge in injection drug users within the country, with subsequent increases in the prevalence of HIV, hepatitis B, and C [14].

People use and inject drugs for many reasons, and interestingly, one drug could be used by different people for several purposes [15]. Inferring from Cox & Klinger, 1988, individuals choose knowingly or unknowingly based on their expected perceived benefits or harm, whether they will engage in substance use or not [16]. In general, people use drugs to boost their energy, to help them deal with restlessness and sluggishness, to aid their feeling of acceptability among their peers, and also to deal with the loss [17, 18].

Some of the reasons for drug use include homelessness, unemployment, peer pressure, working in close contact with drugs at their place of work, and familiarity from overexposure to drug injections being performed by others, leading to their altered views and reduced moral standards of the inappropriate nature of drug injection [19].

Despite the perceived benefits of drug use and injection by those who practice it, it poses a great deal of risk and effect, not only to those who engage in them but also to the communities in which they reside. Such effects include motor accidents, violence, health risks, toxicity and poisoning from excessive doses, financial constraints in seeking healthcare to managing adverse health outcomes, effects on social norms and values, reduced effectiveness and efficiency at work, increased crime rates, as well as deaths [20, 21].

It has been advocated that to facilitate the prevention or reduction in the spread of HIV, HBV, and HCV infection from drug use and injection, harm reduction programs, including needle and syringes, opium substitution therapy, and vaccination programs should be encouraged as well as the effects of sharing injection paraphernalia [22, 23]. The objective of this study was to provide an overview of the prevalence of HIV, HCV, and HBV and their corresponding determinants among a population of drug users and injectors in Ghana.

## 2. Methods

### 2.1. Stakeholders' and Consultative Meetings

The study team organized meetings with relevant stakeholders across four (Greater Accra, Ashanti, Western, and Northern) selected regions and constituted a committee advisory group to help in the successful implementation of the study. From these consultative meetings, communities were selected from each of the regions and included in the assessment.

### 2.2. Study Design, Data Collection, and Sample Size Determination

This assessment was a cross-sectional survey of PWID and PWUD from four (4) regions in Ghana, ensuring adequate representation of the diverse categories of drug users and injectors population across the regions. Communities within regions where the study was carried out were selected purposively with the help of the community advisory group. A quantitative questionnaire was used to solicit information from the respondents and included questions on sociodemographic, sexual behaviour, substance use, and biological characteristics. Furthermore, the prevalence of HIV, HBV, and HCV among PWID and PWUD was determined using blood samples. Before the tests were conducted, participants were first taken through counseling about the relevance or importance of the test and the potential implications if the results of the test were positive. After counseling, blood samples (30uL) were taken for each of the tests. For the HIV test, the Laboratory Technicians used three different types of tests, that is, first response followed by oral quick and then SD bioline as a confirmation test of positivity. For both hepatitis B and C, they used SD bioline. All those who were confirmed positive for any of the three viruses were again counseled and referred to a clinic for treatment if he/she was already not enrolled. All data collectors were trained in data collection procedures for conducting quantitative data, maintaining confidentiality, and appropriately handling and storage of data. The modified Cochran's formula that incorporates the design effect, stratification, and nonresponse rate were used to calculate the sample size for the study. The total number of participants (sample size) required for the study was 303, based on a two-tail hypothesis with an alpha level of 5%.

### 2.3. Sampling Approach (Respondent Driven Sampling)

Respondent-driven sampling (RDS) was used to enroll the PWUD and PWID population in the study. In using the RDS approach, the study team, via key informants and the Community Advisory Board members, identified seeds, and these seeds were recruited or selected from the target population. The seeds that were selected were interviewed, and they formed wave zero (0) of the sample selection. The seeds were recruited, taking into cognizance PWUD, PWID, and female respondents. All selected seeds were given three sets of coupons. They were trained at the center on how to administer the coupons to persons within their social network who were either PWIDs or PWUDs and then refer them to the study location. This recruitment approach then turned into “waves.” The first set of participants who were referred to the study staff then became the first recruits. These first recruits were also trained, and three coupons were given to each and asked to recruit their peers. Each cycle of recruitment and participation added an additional sampling wave. Those who successfully participated in the study were paid for transportation. The transportation cost was necessary because all those who were recruited had traveled to the study venue to participate. All referred participants were screened for eligibility.

### 2.4. Data Analysis

Analysis was conducted using Stata version 17 and RDSAT version 7.1.46 software. RDSAT software was used to calculate individual data weights. Analysis of the quantitative data was done descriptively and presented in the form of medians and proportions (percentages). Inferential analysis was carried out using a Bayesian generalized linear model via the binomial family of distributions under the logit link function between the outcomes, prevalence of HIV, hepatitis B, and hepatitis C, and the predictors; socioeconomic, demographic, and sexual behaviors. All variables that showed a significant relationship with the outcomes of interest and variables that have been reported to be significant predictors of their corresponding outcomes in the literature were entered and used to obtain the adjusted posterior odds ratios (OR) and their credible intervals (CrIs). All regression model parameters were assigned relatively informative priors following the normal and Cauchy distributions with different parameter values. Several sensitivity analyses via specifications (six models) of different priors were established and fitted for each outcome and its corresponding predictor variables. The six models for each outcome based on the assessment of the sensitivity of the prior were compared and the best model was selected for final analysis using Bayesian deviance information criteria, Bayes factor, the marginal likelihood, which was computed via the Laplace-Metropolis approximation and the posterior probabilities. The prior that was used for the prevalence of HIV as an outcome were Gaussian (0, 5) for the logistic distribution constant and Gaussian (0, 0.5) for the model coefficients. Those for the prevalence of HCV were Gaussian (0, 10) for constant and Gaussian (0, 2.5) for the coefficient, while the prevalence of HCV was Cauchy (0, 0.5) and Cauchy (0, 2.5). During the running of the model, 2 chains were specified with 12500 simulations, a thinning of 10, and a burn-in of 2500. Convergence diagnostics were carried out using trace plots, autocorrelation plots, histograms, as well as the Gelman–Rubin convergence rule (Rc < 1.1). All analyses were adjusted for weight. Statistical analyses were completed using StataCorp. 2021. Stata 17 Base Reference Manual (College Station, TX: Stata Press).

## 3. Results

The study engaged drug users and injectors (323) from the Greater Accra, Ashanti, Western, and Northern regions of Ghana. The median age of the participants was 37.0 (IQR: 28–44) years. The median ages at which respondents started to use and inject drugs were 20.0 (IQR: 18.0–26.0) years and 22.0 (IQR 18.0–30.0) years, respectively. The percentages of males and females in the study were 86.1% and 13.9%, respectively. Most of the respondents were JHS leavers (39.7%), followed by those who had at least a Senior Secondary education or higher qualification, with the least (2.3%) of participants who do not have any form of formal education. Participants who were single and never married were 57.0%, and those who had either separated, divorced, or widowed were 15.5%, with the rest being married. Thirty-four percent lived with their sexual partners, and only 0.7% of participants were men who slept with men (Table 1).

### 3.1. Prevalence of HIV, HBV, and HCV among Users and Injectors

The overall prevalence of HIV, HBV, and HCV infection in the study was 2.5%, 4.6%, and 5.9%, respectively. The prevalence of HIV, HBV, and HCV among drug users was 2.5% (95% CI: 0.7%–4.2%), 4.1% (95% CI: 1.8%–6.2%) and 6.7% (95% CI: 3.9%–9.4%) respectively. There was no statistically significant difference between the prevalence of HIV and HBV among noninjectors compared to injectors. For HCV, however, there was a statistically significant increased prevalence among noninjectors versus injectors 6.7% and 0.1% (*p* = 0.002), respectively. By their gender, the prevalence of HIV was highest among females (12.3%) as opposed to males (0.5%). Age was associated (*p* = 0.031) with the prevalence of the hepatitis B virus. We observed that 11.9% of participants had both HBV and HCV, but there were no coinfections among HIV, HBV, and HCV.

### 3.2. Substance Use

Most drug users started using drugs between the ages of 20 and 29 years (37.8%), 35.5% started using drugs before the age of 20 years. Respondents who started injecting drugs at an age of less than 20 years were (29.6%) years (Table 1). Approximately, 30% of drug users used their drugs three times a day and 53.9% did so 4 or more times per day. About 91% and 70.9% of the respondents had, respectively, used and injected drugs within the last 7 days of the study (Table 2).

Of all respondents, 14.0% were drug injectors and 2.7% had used or injected drugs while in prison. Majority (82.1%) of drug injectors were introduced to injecting drugs by their friends and acquaintances. Respondents who engaged in both “snorting, inhaling or swallowing” and “smoking” the drugs were 16.0% (*p* < 0.001) , and those who engaged in both “smoking” and “injecting” the drugs were 7.7% (*p* < 0.001) (Table 3).

### 3.3. Sources of Substances Used by Users and Injectors

Drug injectors in the study sourced their syringes mainly from “pharmacy or chemical shops or stores” (57.4%) and “pharmacy workers or drug vendors” (20.7%). More than 69.0% of drug injectors reused their needles and the main reasons were because of their perceived high cost (29.9%) and the difficulty in accessing needles (26.9%). A large proportion of injectors (67.4%) never shared needles with another person. However, 12.7% shared their needles half of the time, and 7.8% shared their needles very frequently (Table 4).

### 3.4. Women Who Use and Inject Drugs

More than half of the women in the study, 62.2% (28/45), were less than 30 years. A higher percentage of women were single (69.9% (31/45)) and separated (20.1% (9/45)). More than 90.0% (42/45) of female drug users had at least primary education, and 62.2% (28/45) were unemployed. Only 2.6% (1/45) of women in the study injected a drug (heroin), but 54.7% (25/45) used two or more drugs together (Table 1). Sixty-six percent (30/45) of females in the study lived with a sexual partner, 45.4% (20/45) had ever received or given money, goods, or gifts in exchange for sex, and 28.9% (13/45) had ever received or given drugs in exchange for sex. About 22% (10/45) had suffered physical or sexual violence in the last 6 months.

### 3.5. Factors Influencing HIV, HBV, and HCV Infection

In the adjusted model, respondents aged 40 years and above have 52% lower posterior odds (POR = 0.48, CrI: 0.18–0.98) of being infected with HIV when compared to respondents in the age group of 19–29 years. Being a Junior High or Middle School graduate significantly protected the respondents (59%) from being HIV infected (POR = 0.41, CrI: 0.14–0.97). Belonging to the general population, compared to being a sex worker, has a statistically significant negative relationship (66%) with being tested HIV positive or infected (POR = 0.34, CrI: 0.12–0.81). There were lower posterior odds (70%) among respondents who have completed Junior High or Middle school as against those who never attended school of being infected with HBV (POR = 0.30, CrI: 0.07–0.84). For HBV infection, a fivefold higher (POR = 5.50, CrI: 1.20–16.16) statistically significant relationship was observed among respondents who are employed on a part-time base compared to those employed full time and participants who operate within the Ashanti region (POR = 0.16, CrI: 0.01–0.57) as against their Greater Accra counterparts, Table 5.

## 4. Discussion

Given the change in global and national priorities following the onset of the Coronavirus disease (COVID-19), pandemic attention has been duly shifted from already existing issues to the imminent matter at hand [24,25]. Among such deferred issues is that morbidities such as HIV, HBV, and HCV existing among drug users and injectors [26–28]. Drug use and injection and their consequences on health have been an issue of public health concern for a long time [29,30]. This study successfully engaged PWUD and PWID, who willingly and effectively contributed to the findings through their responses and referrals. This is the first study to be conducted in four regions across the three ecological zones to examine the prevalence and determinants of HIV, HBV, and HCV among PWUD and PWID in Ghana.

Most of the respondents in this study were males. However, recent studies suggested ever-increasing numbers of female drug users, thus closing the gender gap between male and female drug users and injectors [31,32]. According to Strashny, there was a significantly higher proportion of males than females engaged in substance use within early adolescence compared to the almost similar proportion of male to female ratio after 24 years of age [32]. There is a need for more attention to be given to gender differences related to the dynamics and effects of substance use, given the increasing participation of women [32–34].

Most drug users and injectors were initiated into practice by their friends, acquaintances, and relatives, which is similar to other findings [35]. Commonly used drugs by first-time drug users in this study included marijuana, crack, and heroin. Findings available suggest similar drugs in addition to methamphetamine, tobacco, cannabis, and others [31, 32, 36]. It was interesting to note that most drug users and injectors engaged in the practice at least once daily, although some respondents went as far as four or more times in a day. This is similar to other findings suggesting that users and injectors use and inject more drugs on a daily basis [31, 37, 38].

Most of the study participants never shared their injection needles or syringes. However, they reused their own needles after they were sourced from pharmacies or drug dealers. The majority of drug injectors (93.0% and 62.0%) hardly shared their needles although they mostly reused them [35, 38–40]. The reuse of needles may be because of the inability of injectors to either purchase or have access to needles. Other reasons include unavailability at the time of injection, peer pressure, and difficulty to walk about due to police disturbances [29].

Women who used and injected drugs in the study were mostly single and educated but unemployed. This is consistent with already existing literature, where it is stated that only 22.8% of female drug users were in some form of full or part-time employment [41]. The only woman injector in this study commonly injected heroin, which is contrary to a study where women were more likely to inject antianxiety medications and methamphetamine [41] and was initiated at 16.0 years. This conforms to a study where males have an early debut of drug use and injection as against females [42]. Close to half of the women in the study had ever exchanged sex for goods or drugs. Previous studies indicated that women traded sex for money or drugs or had received drugs for their first injection as a gift and thereafter mostly engaged in sexual relations with their partners before or after drug injection [42, 43].

More women had HIV in the study compared to their male counterparts is found in the literature that HIV is generally higher among women. This may be exacerbated due to sexual abuse [30, 42]. In a related study, women had more multiple sexual partners as compared to men [44]. Other reasons include gender inequalities such as decent employment for men than women, lower literacy levels, and women's restricted opportunity to have some level of control over resources. It was further stated that when women were less financially empowered, they happened to rely more on their male partners for survival, thus being at their mercy. These women may have even lacked the skills to negotiate safe sex due to their early sexual debut, which predisposes them to such infections at an early stage [45–48]. More men than women were found to be infected with hepatitis B and C viruses. This is similar to other findings, where hepatitis, in general, is higher in men than women [49–51].

Age was a significant predictor of HIV. The higher the age, the less likely one was to be infected with HIV. This was contrary to other findings suggesting that the higher the age, the higher probability of HIV infection [3]. Education significantly predicted HIV and HBV status in this study. This was confirmed by studies where significant associations were found between having less than high school education and being more likely to have HIV and HBV [3, 10]. Other studies also confirmed the positive effects of education on HIV status. They argue that the more individuals obtain education, the more aware they become of risky behaviors and their consequences. More educated individuals were more likely to protect themselves since they were more knowledgeable of precautionary measures against these diseases [52–54]. There was a significantly lower prevalence of HIV among people who were identified to be in the general population. Those who reported as sex workers or men who have sex with men had higher odds of being HIV positive. The rate of infection among sex workers and men who have sex with men (MSM) was very alarming, given the number of sexual partners involved and the risky sexual behavior [3, 55–58]. Employment status was another significant predictor of HBV status. Those who were employed part-time were five times more likely than those who were employed full-time to be infected with HBV. A past study, on the contrary, reported that most respondents who were unemployed experienced blood-borne infections of which hepatitis B was a part [10].

### 4.1. Recommendations

1. Full harm reduction programs, including NSP and OST, should be intensified among drug injectors to help reduce their chances of needle sharing and reuse.
2. Continuous awareness and education on the potential for risky sexual behaviors among drug users should be encouraged, in addition to interventions that can help curb such practices of risky sexual behavior.
3. Rehabilitation programs to help people with substance use disorder who are willing to quit the practice.

## 5. Conclusion

Findings from this study suggest that most drug users and injectors had a reasonable level of education and fair level of employment. Most were not married, but a third lived with their sexual partners. The majority of respondents belonged to the general population, where they were not sex workers or men who slept with men. Commonly used and injected drugs included heroin, crack, cocaine, and tramadol, and these were used or injected daily from one to more than four times a day. They were mostly introduced by friends, relatives, and acquaintances. Education, literacy, and not being a member of a key population protected respondents from HIV, hepatitis B, and C infections.

## Figures and Tables

**Table 1:** Distribution of respondents' (drug users and injectors) characteristics according to their HIV, HCV, and HBV status.

	Unweighted	Weighted	HIV	HCV	HBV
	323 (100) n(%)	323 (100) *n*(%)	n(%)	n(%)	n(%)
Factor			%	%	%

Age of participant, median (IQR)	36 (28, 43)	37 (28, 44)	39 (28, 43)	44 (34, 54)	40 (32, 50)

Age group of participants					
19–29 years	91 (28.2)	90 (27.7)	30.3	17.0	13.7
30–39 years	112 (34.7)	113 (35.1)	24.5	29.0	32.7
40–49 years	75 (23.2)	71 (22.1)	45.3	21.3	21.6
>49 years	42 (13.0)	47 (14.6)	0.0	32.7	25.5
Nonresponse	3 (0.9)	2 (0.5)	0.0	0.0	6.5

Highest level of education					
Never attended school	7 (2.2)	7 (2.3)	14.0	5.2	9.8
Primary	75 (23.2)	81 (25.0)	15.1	39.4	23.5
JHS/middle school	132 (40.9)	128 (39.7)	29.1	31.0	34.0
SHS/SSS/Voc./Tech.	91 (28.2)	96 (29.7)	41.8	18.4	21.6
Tertiary	18 (5.6)	11 (3.4)	0.0	5 .9	11.1

Employment status					
Employed full-time	125 (38.7)	116 (36.0)	36.1	49.8	13.7
Employed part-time	64 (19.8)	78 (24.1)	30.1	23.6	55.0
Full-time student	3 (0.9)	2 (0.6)	0.0	0.0	0.0
Retired	2 (0.6)	2 (0.7)	0.0	0.0	0.0
Unemployed	129 (39.9)	125 (38.6)	33.8	26.6	31.4

Marital status					
Single, never married	196 (60.7)	184 (57.0)	78.0	62.5	52.3
Married	85 (26.3)	89 (27.4)	3.5	22.6	21.6
Separated/divorced	17 (5.3)	24 (7.5)	18.6	15.0	19.6
Widowed	25 (7.7)	26 (8.0)	0.0	0.0	6.5

Currently living with sexual partner					
Yes	118 (36.5)	110 (34.1)	32.6	15.6	39.2
No	205 (63.5)	213 (65.9)	67.4	84.4	60.8

Monthly income					
Less than 200 GHC	70 (21.7)	66 (20.3)	36.1	11.2	27.5
200 to 500 GHC	78 (24.2)	77 (23.8)	39.4	41.7	29.4
500 to 1000 GHC	79 (24.5)	84 (26.0)	0.0	28.6	7.8
1000 to 2000 GHC	59 (18.3)	60 (18.7)	17.5	18.6	11.8
Above 2000 GHC	23 (7.1)	21 (6.5)	7.0	0.0	15.7
Don't know	6 (1.9)	8 (2.5)	0.0	0.0	7.8
Refuse to answer	8 (2.5)	7 (2.2)	0.0	0.0	0.0

How respondents identify self					
SW	15 (4.6)	13 (3.9)	29.1	0.0	0.0
MSM	2 (0.6)	2 (0.7)	0.0	0.0	0.0
General population	303 (93.8)	305(94.4)	70.9	100.0	100.0
Refuse to answer	3 (0.9)	3 (1.1)	0.0	0.00	0.0

Age at start of using of drugs, median (IQR)	20 (18, 25)	20 (18, 26)	16, 28	22, 33	17, 24

Age group at start of using drugs					
<15 years	14 (4.3)	14 (4.4)	0.0	0.0	0.0
15–19 years	107 (33.1)	100 (31.1)	37.3	17.9	40.0
20–24 years	82 (25.4)	83 (25.8)	15.1	21.1	13.7
25–29 years	40 (12.4)	39 (12.0)	30.1	26.8	9.8
>30 years	51 (15.8)	55 (16.9)	17.5	28.3	7.7
Non-response	29 (9.0)	32 (9.7)	0.0	6.0	38.6

Age at start of injecting drugs, median (IQR)	23.5 (19, 30)	22 (18, 30)	16, 16	20, 20	20, 35

Age group at start of injecting drugs					
<15 years	3 (5.8)	3 (5.6)	0.0	0.0	0.0
15–19 years	12 (23.1)	12 (23.9)	100.0	0.0	0.0
20–24 years	12 (23.1)	13 (25.0)	0.0	100.0	40.0
25–29 years	11 (21.2)	9 (16.6)	0.0	0.0	0.0
>30 years	14 (26.9)	15 (28.9)	0.0	0.0	50.0

Regions					
Greater accra	108 (33.4)	134 (41.5)	37.1	94.1	41.2
Ashanti	90 (27.9)	76 (23.5)	45.4	0.0	16.3
Western	86 (26.6)	93 (28.8)	17.47	6.0	39.2
Northern	39 (12.1)	20 (6.1)	00.00	0.0	3.3

**Table 2:** Distribution of respondents' who use drugs for HIV, hepatitis B, and C infection.

	Unweighted	Weighted	HIV	HCV	HBV
	*n* (%)	*n* (%)	*n*(%)	*n* (%)	*n* (%)
Factor	323 (100)	323 (100)	8 (100)	19 (100.0)	15(100.0)

Age group at start of using drugs					
<15 years	14 (4.3)	14 (4.4)	0	0	0
15–19 years	107 (33.1)	100 (31.1)	37.28	17.89	33.99
20–24 years	82 (25.4)	83 (25.8)	15.14	21.11	13.73
25–29 years	40 (12.4)	38 (12.0)	30.11	26.78	9.8
>30 years	51 (15.8)	54 (16.9)	17.47	28.27	3.92

Drug used at first time^(M)^					
Cocaine	27 (8.4)	22 (7.0)	29.12	0	0
Crack	33 (10.2)	38 (11.9)	38.44	28.27	17.65
Heroin	44 (13.6)	56 (17.4)	0	19.84	3.92
Marijuana	158 (48.9)	155 (48.2)	32.44	32.55	54.25
Campucheas (heroin mixed with marijuana	1 (0.3)	1 (0.3)	0	0	6.54
Cocktail (marijuana + heroin)	7 (2.2)	7 (2.1)	0	13.39	0
Tramadol	25 (7.7)	13 (4.1)	0	0	0
Alcohol	2 (0.6)	2 (0.9)	0	0	0
Cigarette	3 (0.9)	3 (1.0)	0	0	0
Prescription drugs	2 (0.6)	1 (0.5)	0	0	0
Refused to answer	21 (6.5)	21 (6.7)	0	5.95	17.65

Ever changed from one drug to the other					
Yes	246 (76.2)	239 (74.2)	51.08	58.34	82.35
No	56 (17.3)	61 (19.2)	48.92	35.71	0
Nonresponse	21 (6.5)	21 (6.7)	0	5.95	17.65

What did it change to?					
Cocaine	76 (30.9)	88 (35.8)	31.93	15.79	34.13
Crack	57 (23.2)	52 (21.5)	0	0	0
Heroin	64 (26.0)	71 (29.2)	45.26	84.21	57.14
Marijuana	10 (4.1)	9 (3.9)	0	0	0
Cocktail	3 (1.2)	2 (0.6)	0	0	0
Crystal meth/methamphetamine	6 (2.4)	3 (1.2)	0	0	4.76
Tramadol	18 (7.3)	13 (5.2)	22.81	0	1.59
Pethidine	3 (1.2)	2 (0.8)	0	0	2.38
Opiates	3 (1.2)	1 (0.4)	0	0	0
Prescription drugs	6 (2.4)	4 (1.5)	0	0	0

Last time drug was used					
Within the last 7 days	299 (92.6)	295 (91.5)	100	94.05	100
More than 7 days to 1 month	3 (0.9)	2 (0.6)	0	0	0
More than 1 month up to 6 months	4 (1.2)	4 (1.1)	0	0	0
More than 6 up and to 12 months	1 (0.3)	1 (0.3)	0	0	0
More than 12 months	16 (5.0)	21 (6.5)	0	5.95	0
Number of times per day drug is used	8 (2.7)	5 (1.6)	0	0	0
1 time/day	39 (12.9)	43 (14.2)	13.98	30.06	18.25
2 times/day	92 (30.5)	92 (30.3)	8.15	48.81	32.54
3 times/day	54 (17.9)	53 (17.4)	31.45	17.18	7.94
4+ times/day	109 (36.1)	110 (36.5)	46.42	3.95	41.27
>4 times/day					

Commonly use two or more drugs together					
No	214 (66.3)	209 (64.7)	63.89	73.22	53.59
Yes	80 (24.8)	83 (25.8)	36.11	20.83	28.76
No response	29 (9.0)	31 (9.5)	0	5.95	17.65

**Table 3:** Unweighted and weighted characteristics of respondents by variable type according to people who inject drugs.

	Unweighted	Weighted
	*n* (%)	*n* (%)
Factor	52 (100)	52 (100)

Age group at start of injecting drugs		
<15 years	12 (23.1)	3 (5.6)
15–19 years	12 (23.1)	12 (23.9)
20–24 years	11 (21.2)	13 (25.0)
25–29 years	14 (26.9)	9 (16.6)
>30 years	12 (23.1)	15 (28.9)

Person who introduced injecting		
A relative or family member	3 (5.9)	4 (8.3)
A person you use drugs with	4 (7.8)	3 (5.7)
A friend/an acquaintance	42 (82.4)	42 (82.1)
A stranger	1 (2.0)	1 (2.6)
Others	1 (2.0)	1 (1.3)

Drug injected at first time^(M)^		
Cocaine	22 (42.3)	27 (51.1)
Crack	9 (17.3)	5 (10.1)
Heroin	8 (15.4)	11 (21.0)
Marijuana	1 (2.0)	0 (0.9)
Crystal meth/methamphetamine	1 (2.0)	0 (0.6)
Tramad	6 (11.5)	4.6 (8.9)
Pethidine	6 (11.5)	4 (8.2)

Ever changed from one injecting drug to the other		
Yes	16 (31.4)	13 (25.8)
No	34 (66.7)	36 (69.9)
Nonresponse	1 (2.0)	2 (4.3)

What did it change to?		
Cocaine	7 (43.8)	8 (52.7)
Crack	4 (25.0)	3 (16.9)
Heroin	2 (12.5)	2 (10.9)
Crystal meth/methamphetamine	1 (6.3)	1 (6.2)
Other	2 (12.5)	2 (13.2)

Last time injected drugs		
Within the last 7 days	37 (71.2)	37 (70.9)
More than 7 days to 1 month	4 (7.7)	3 (6.1)
More than 1 month up to 6 months	5 (9.6)	4 (6.8)
More than 6 up and to 12 months	2 (3.9)	3 (6.5)
More than 12 months	4 (7.7)	5 (9.8)

Commonly injected drugs		
Cocaine	23 (45.1)	28 (55.2)
Crack	9 (17.7)	6 (12.4)
Heroin	7 (13.7)	8 (14.9)
Crystal meth/methamphetamine	1 (2.0)	0 (0.7)
Tramadol	6 (11.7)	5 (9.1)
Pethidine	4 (7.8)	3 (6.9)
Other	1 (2.0)	0 (0.9)

Number of times per day drug is injected		
1 time/day	14 (27.5)	0 (27.6)
2 times/day	13 (25.5)	13 (25.2)
3 times/day	9 (17.7)	9 (17.5)
4+ times/day	15 (29.4)	15 (29.8)

Commonly use two or more drugs together		
No	31 (67.4)	32 (69.6)
Yes	15 (32.6)	14 (30.4)

**Table 4:** Unweighted and weighted characteristics of respondents by variable type according to people who inject drugs.

	Unweighted	Weighted
	*n* (%)	*n* (%)
Factor	52	52

Source of needles/syringes in the last 6 months		
Pharmacy/chemist/drug store/store/another store	29 (61.7)	27 (57.4)
Market place or street vendor	2 (4.3)	1 (2.7)
Pharmacy worker or drug vendor	8 (17.0)	10 (20.7)
Sex partner, friend, acquaintance, relative	3 (6.4)	2 (5.3)
Drug dealer or other drug users	4 (8.5)	4 (9.0)
Don't know	1 (2.1)	2 (4.9)

Sterile needles and syringes available when needed		
Yes	44 (86.3)	41 (80.6)
No	5 (9.8)	5 (10.8)
Don't know	2 (3.9)	4 (8.7)

Pays for the needles		
Yes	44 (88.0)	46 (92.4)
No	6 (12.0)	4 (7.6)

Pay to be injected		
Yes	16 (31.4)	18 (35.5)
No	35 (68.6)	33 (64.5)

How often is a new sterile needle used in the last 6 months		
Never	2 (3.9)	2 (3.8)
Rarely	5 (9.8)	5 (9.0)
Half of the time	6 (11.8)	6 (12.0)
Most of the time	9 (17.7)	7 (14.7)
Always	29 (56.9)	31 (60.5)

Reasons for not using a new needle or syringe always		
Not available	2 (9.1)	2 (8.9)
Difficult to find	6 (27.3)	6 (26.9)
Expensive	7 (31.8)	7 (30.0)
Peer pressured to share	3 (13.6)	5 (20.9)
I reuse my own needle	4 (18.2)	3 (13.4)

When you inject, do you do it: alone, or with a friend, drugs dealer, assistant drug dealer		
Alone	25 (49.0)	27 (52.5)
A friend	19 (37.3)	18 (35.1)
Drugs dealer	5 (9.8)	4 (8.5)
Assistant drug dealer	1 (2.0)	1 (1.3)
Don't know	1 (2.0)	1 (2.6)

In the last 6 months, how often did you use needles that someone else had already injected with		
Never	38 (74.5)	34 (67.4)
Rarely	2 (3.9)	3 (6.5)
Half of the time	6 (11.8)	6 (12.7)
Most of the time	3 (5.9)	4 (7.8)
Always	6 (11.7)	1 (1.3)
Don't know	1 (2.0)	2 (4.3)

Shared needle with other person in the past 6 months		
Not shared	26 (57.8)	23 (52.2)
Shared	13 (28.9)	14 (31.6)
Refused to answer	6 (13.3)	7 (16.2)

Shared an instrument with another person in the past 6 months		
Not shared	24 (54.6)	22 (50.0)
Shared	20 (45.5)	22 (50.0)

Venue or location drug is commonly injected		
Own house	13 (26.5)	15 (30.6)
House of someone else	3 (6.1)	4 (7.3)
House of dealer	3 (6.1)	3 (6.2)
Abandoned building	2 (4.1)	2 (4.3)
Street (ghetto)	24 (49.0)	23 (46.2)
Other places	4 (8.2)	3 (5.5)

How often are needle/syringe reused before thrown out		
Very often	13 (25.5)	12 (23.9)
Often	7 (13.7)	8 (15.0)
Not so often	13 (25.5)	15 (29.9)
Never	18 (35.3)	16 (31.3)

**Table 5:** Crude and adjusted posterior medians (odds ratios) and their credible intervals for the prevalence of HIV, HCV, and HBV.

	Crude HIV	Crude HCV	Crude HBV	Adjusted HIV	Adjusted HCV	Adjusted HBV
Factor						

Age group of participants						
19–29 years	1.00	1.00	1.00	1.00	1.00	1.00
30–39 years	0.97 (0.30–2.29)	0.99(0.35–2.19)	1.65 (0.37–4.86)	0.83 (0.25–1.95)	1.05 (0.36–2.32)	1.26 (0.28–3.73)
40+ years	0.95 (0.31–2.28)	1.27(0.47–2.73)	1.59 (0.36–4.60)	**0.48 (0.18–0.98)**	1.17 (0.40–2.69)	1.84 (0.37–5.63)

Highest level of education						
Never attended school	1.00	1.00	1.00	(1.00	1.00	1.00
Primary	1.19 (0.37–2.92)	1.59(0.53–3.72)	0.65 (0.71–2.59)	1.12 (0.46–2.28)	1.55 (0.51–3.64)	0.52 (0.79–1.82)
JHS/middle school	0.68 (0.20–1.74)	0.68(0.21–1.58)	0.67 (0.09–2.46)	**0.41 (0.14–0.97)**	0.69 (0.22–1.62)	**0.30 (0.07–0.84)**
Secondary school or higher	0.94 (0.29–2.28)	0.78(0.26–1.91)	1.21 (0.17–4.42)	0.67 (0.24–1.53)	0.79 (0.26–1.86)	1.52 (0.29–4.83)

Employment status						
Employed full-time	1.00	1.00	1.00	1.00	1.00	1.00
Employed part-time	1.16 (0.35–2.88)	1.38(0.49–3.04)	**3.85 (1.39–10.08)**	1.25 (0.40–3.09)	1.36 (0.49–2.97)	**5.50 (1.20–16.16)**
Unemployed	1.13 (0.37–2.61)	0.69(0.25–1.49)	1.22 (0.27–3.69)	1.03 (0.36–2.32)	0.67 (0.24–1.44)	2.13 (0.47–5.99)

Marital status						
Single, never married	1.05 (0.34–2.63)	1.20(0.44–2.73)	1.11 (0.24–3.53)	0.82 (0.31–1.84)	1.09 (0.41–2.37)	2.38 (0.49–7.66)
Married	0.74 (0.19–1.92)	1.32(0.43–3.18)	0.97 (0.15–3.30)	0.53 (0.15–1.27)	0.82 (0.23–2.05)	2.10 (0.39–7.07)
Separated/divorced/Widowed	1.00	1.00	1.00	1.00	1.00	1.00

Currently living with sexual partner						
Yes	1.00	1.00	1.00	1.00	1.00	1.00
No	**0.04 (0.01–0.07)**	1.77(0.71–3.93)	0.57 (0.17–1.38)	0.99 (0.36–2.24)	1.75 (0.67–3.92)	0.54 (0.14–1.29)

How respondents identify self						
SW	1.00	1.00	1.00	1.00	1.00	1.00
MSM	—	—	—	—	—	—
General population	0.38 (0.11–1.02)	—	—	**0.34(0.12–0.81)**	—	—

Injecting drugs						
Yes	0.94(0.25–2.37)	0.71(0.21–1.68)	1.77(0.36–4.80)	1.23(0.33–3.28)	0.70(0.20–1.73)	1.47(0.18–5.29)
No	1.00	1.00	1.00	1.00	1.00	1.00

Age group at start of using drugs						
Less than or equal to 19 years	0.96 (0.29–2.28)	0.71(0.24–1.62)	1.24 (0.22–4.06)	0.47 (0.17–1.06)	1.43 (0.53–3.15)	2.09 (0.41–6.82)
20–29 years	1.36 (0.42–3.37)	1.07(0.39–2.39)	1.30 (0.23–4.28)	0.76 (0.32–1.52)	1.57 (0.49–3.76)	1.99 (0.28–7.22)
30+ years	1.00	1.00	1.00	1.00	1.00	1.00

Regions						
Greater accra	1.00	**3.37(1.26–7.57)**	1.00	1.00	1.00	1.00
Ashanti	1.35 (0.44–3.14)	—	0.80 (0.25–1.81)	2.02 (0.70–4.5)	—	**0.16 (0.01–0.57)**
Western	0.91 (0.28–2.19)	—	1.45 (0.49–3.23)	1.67 (0.56–4.13)	—	2.02 (0.55–5.13)
Northern	—	1.00	1.26 (0.18–0.78)	—	—	0.83 (0.55–5.13)

## Data Availability

The datasets generated and/or analyzed during the current study are not publicly available but are available from the corresponding author on request.
